# Evaluation of cranial tibial translation in dogs: Diagnostic accuracy of radiographic method using a simple device

**DOI:** 10.1371/journal.pone.0228621

**Published:** 2020-02-11

**Authors:** Adolfo Maria Tambella, Luca Omini, Anna Rita Attili, Cecilia Vullo, Stefano Martin

**Affiliations:** 1 University of Camerino, School of Biosciences and Veterinary Medicine, Matelica (MC), Italy; 2 Clinica Veterinaria Omini Luca, Chiaravalle (AN), Italy; University of Memphis, UNITED STATES

## Abstract

A hand-made, radiolucent, custom-designed device having a mobile and a non-mobile platforms was used to objectively quantify the *in vivo* cranial tibial translation, in order to assess the functional status of cranial-cruciate-ligament (CrCL) in dogs. The hypothesis was that changes in CrCL integrity would result in detectable changes in tibial translation. To validate the diagnostic method, data from injured (PA, n = 32), contralateral (CO, n = 32) and healthy stifles (HE, n = 32) were compared. Normalized tibial translation (Δ_N_) of each stifle was measured in medio-lateral radiographic projection obtained before and during standard thrust force application, in PA (43.59±12.97%), CO (20.32±6.69%) and HE (12.22±3.77%). Comparing PA with HE and CO (Δ_N_ cut-off value: 29.73%), diagnosis could be issued with very high probability. Comparing HE with CO (Δ_N_ cut-off value: 14.80%), high performance was obtained. The translator device could be a useful tool to objectively quantify the *in vivo* tibial translation in dogs with CrCL rupture, before surgery and during post-operatory follow-up.

## Introduction

The major role of the cranial cruciate ligament (CrCL) to prevent cranial tibial translation is well established by scientific literature [1–4].

Physical and radiographic examination techniques are commonly used to diagnose CrCL deficiency. Clinical detection of tibial translation by cranial drawer test and tibial compression test can aid revealing stifle joint instability as a result of CrCL injury. However, diagnosis of CrCL insufficiency using these tests is subjective and difficult to quantify accurately [5,6].

In order to thoroughly assess the joint stability as well as joint stabilization after surgery, it is imperative to compare joint stability between and within subjects over time. There are several descriptions of radiographic techniques to assess translational stifle stability and CrCL integrity in dogs, but they do not include a specific joint angle or controlled force application to accomplish tibial translation [7,8]. Lopez described a radiographic technique to measure the tibial translation in intact, partially and completely ruptured CrCL [9]. Kim et al. quantified cranial tibial subluxation by means of implantation of radio-opaque markers at the femoral and tibial attachments of the CrCL [10], but this may not be practical to implement in clinical patients. Bielecki et al. measured tibial subluxation in normal cadavers after ligament transection and during gradually increasing tibial thrust by manual flexing the hock joint [11]. Castaneda et al. used normal cadaveric canine pelvic limbs to investigate a measurement technique for assessing the degree of cranial tibial displacement, at varying degree of stifle flexion, in intact, partially and completely transected conditions while manually flexing the tarsal joint [6]. A special joint-testing machine was used *ex vivo* to evaluate radiographically and electromagnetically the passive laxity of normal canine stifle before and after cutting the CrCL, and also after surgical treatment [12]. Recently, Srisuwanporn et al. examined the accuracy of a new stress radiographic device for measuring tibial translation in diagnosing anterior cruciate ligament tear in people [13]. Plesman et al. described radiographic landmarks for measurement of cranial tibial subluxation in normal canine pelvic limb before and after transection of cranial cruciate ligament [14]. Other studies investigated potential predictive value for canine CrCL rupture from radiographic risk factors, such as the severity of synovial effusion and osteophytes [15], the status of infrapatellar fat pad [16], or the conformation factors of pelvic limb [17].

In order to assess the integrity of CrCL, this study was designed to objectively quantify the *in vivo* cranial canine stifle translation using a simple, hand-made, radiolucent translator device keeping fixed the joint angle during the thrust. The hypothesis was that changes in CrCL integrity would result in detectable changes in tibial translation. If the hypothesis is confirmed, the radiographic method using this simple instrument could be included, after validation, in the evaluation protocols of orthopaedic studies, potentially representing a concrete help for researchers and clinicians in the specific area.

## Materials and methods

In this prospective study, the principles of STARD guidelines (Standards for Reporting of Diagnostic Accuracy Studies) were followed (STARD checklist in S1 Table) [18,19].

The protocol was approved by the Institutional Ethics Committee for the protection of animals used for experimental or other scientific purposes of the University of Camerino in accordance with Good Scientific Practice guidelines and national legislation (Approval No. 122014).

### Inclusion criteria

The study took place at the Veterinary Teaching Hospital, School of Biosciences and Veterinary Medicine, University of Camerino between May 2017 and December 2018.

Adult dogs of all breeds and gender, weighing more than 15 kg were included in the study.

All the animals had to be assigned to American Society of Anesthesiologists (ASA) categories I or II on the basis of a thorough physical and hematological evaluation. Exclusion criteria were animals in ASA categories III to V, pregnant or lactating bitches, and presence of other orthopedic problems beyond the complete CrCL rupture.

For each dog enrolled in the study, informed owner consent was obtained.

### Test groups

Three groups of stifles were included in the study to validate the diagnostic method: canine stifles with intact CrCL (healthy, Group HE); canine stifles with naturally occurring, unilateral, complete CrCL rupture (pathological, Group PA); contralateral stifles of affected dogs (contralateral, Group CO). Group PA had to show clinical signs related to the CrCL rupture, positive drawer sign both in extension and flexion, and positive cranial tibial thrust tests, with no radiographic signs of degenerative joint disease (DJD) or with a degree of DJD not exceeding the mild level [20]; Groups HE and CO did not have to show clinical signs or positive tests. Group PA had the diagnosis of CrCL rupture confirmed subsequently by inspection of the ligament at arthrotomy during a therapeutic surgical procedure. Group HE consisted of a consecutive series of healthy dog anaesthetized for reason other than for investigation of stifle lameness; they were with no sign of stifle abnormality, as evaluated by normal physical examination and confirmed by orthopaedic and radiographic examinations.

### Anaesthesia procedure

Food but not water was withheld for 10 hours before anesthesia. Following determination of baseline data, a catheter was aseptically placed into a cephalic vein and dogs were premedicated with an endovenous (IV) injection of 3 μg/kg dexmedetomidine (Dexdomitor; Orion Pharma, Italy) and 0.2 mg/kg methadone (Semfortan; Dechra, Italy) mixed in the same syringe. Ten minutes after the premedication, anesthesia was induced with IV propofol (Propovet; Vetfol, Italy) to effect before intubation. The dogs were connected to a small animal rebreathing circuit and anesthesia was maintained with isoflurane (Isoflo; Zoetis, Italy) in 100% oxygen.

After performing the radiographic study, anesthesia was extended for surgery using isoflurane. Immediately before surgery 1.0 mg/kg of tramadol (Altadol; Formevet, Italy), diluted in distilled water to a final volume of 0.22 ml/kg, was administered epidurally at the lumbo-sacral space. During surgery the dogs received a loading dose of 2 mg/kg lidocaine (Lidocaine 2%; Ati, Italy) followed by a constant rate infusion of 100 μg/kg/minute administered throughout anesthesia and surgery.

### Evaluation procedure

A handmade, radiolucent, custom-designed device having a mobile and a non-mobile platforms was used in each dog to objectively quantify the *in vivo* cranial tibial translation, keeping 135° of joint angle, which is typically considered as the average standing angle of the canine stifle [21–23] and as performed by Lopez and collaborators [9].

**Fig 1:**
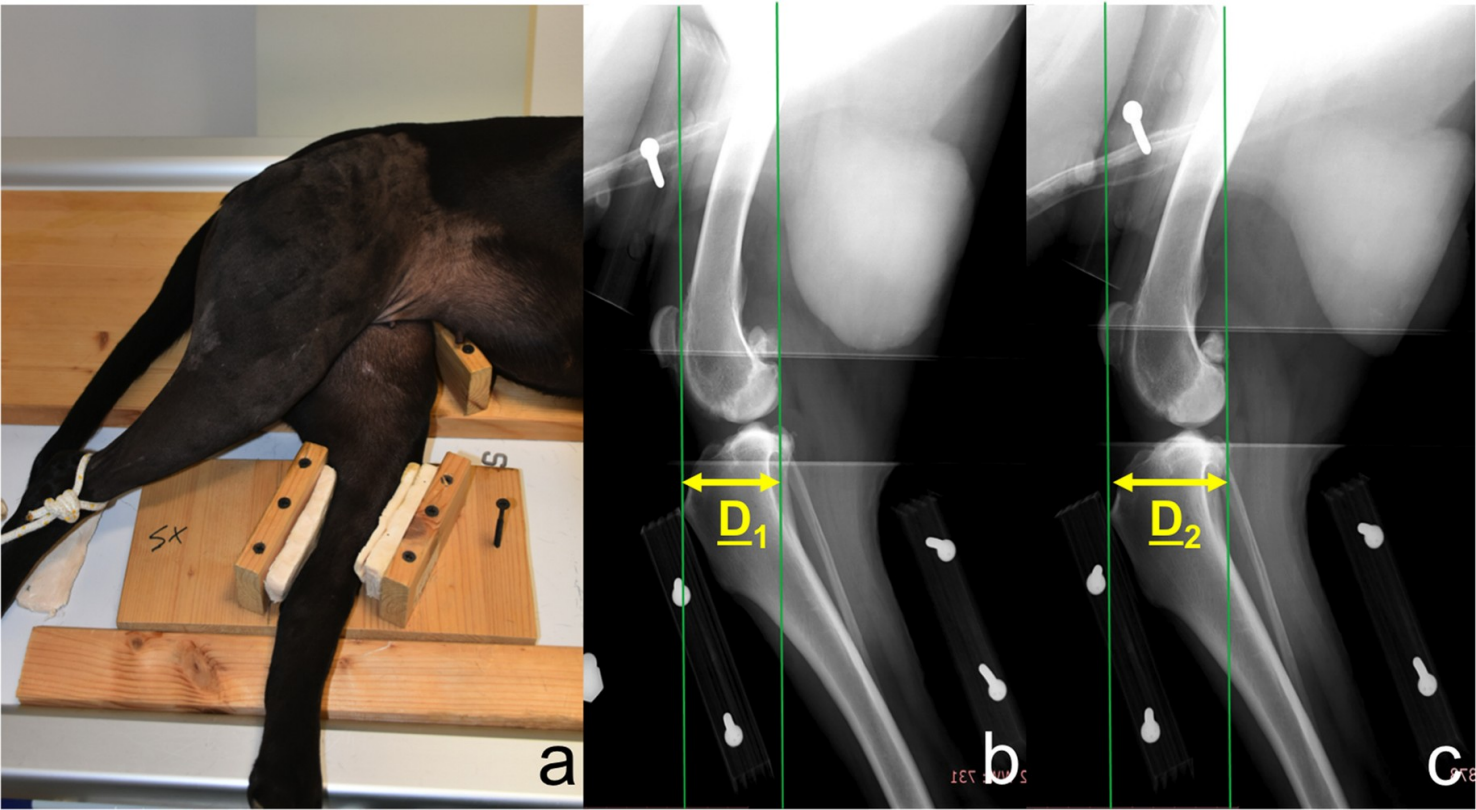
Positioning of dog on the translator device during the test (a); medio-lateral radiographic projections of the stifle using the translator device before (b) and during standard force application (c) in order to thrust the tibia cranially. Green vertical lines represent reference lines for measurements. Yellow arrows indicate the distance between reference lines before (D_1_) and during force application (D_2_).

Dogs under general anaesthesia were positioned in lateral recumbence with the hind limb on the translator device: femur was allocated on the immobile platform while tibia was secured to the mobile platform with polystyrene blocks in the respective housings (Fig 1A).

A force was manually applied to the tibial platform in caudal direction to limit the risk of spontaneous cranial positioning of the tibia at rest, obtaining a zero point for the translation measurement.

Medio-lateral radiographic projections were obtained in each stifle, before and during standard force application (49 N, horizontal plane, cranial direction) to the mobile tibial platform, in order to thrust the tibia cranially. The central radiographic beam was consistently centered on each stifle.

The standard force of 49 N used in this study was the result of the sum of the force already used by Lopez and collaborators (44.5 N) [9] and the average static sliding friction of the mobile tibial platform on the radiologic table surface (4.5 N) previously measured in two dogs weighing 15 and 45 kg. The force was measured using a digital dynamometer (mini crane scale) and it was applied and maintained during the execution of the radiographs by using a weight corresponding to the force required to perform the test.

The cranial tibial translation was measured digitally on each radiographic image (Fujifilm, FCR-Capsula-V-View). Two vertical, parallel lines were drawn perpendicularly to the vector force and tangent to the apex of tibial crest or the caudal edge of femoral condyles respectively. The distance between these two lines was measured in mm before (D_1_ in Fig 1B) and during force application (D_2_ in Fig 1C).

The difference between D_2_ and D_1_ expressed the absolute tibial translation (Δ_S_) [Δ_S_ = D_2_-D_1_]. Δ_S_ was normalized to obtain data regardless of dog’s size. Tibial width (TW) was measured in mm in medio-lateral projection on the distal portion of the tibial crest, perpendicularly to the long axis of the tibia. Normalized tibial translation (Δ_N_) was obtained as relative percentage of TW [Δ_N_ = Δ_S_/TW×100] as performed in other studies [9,24,25].

Two operators, who did not come into contact with dogs and were unaware of the clinical condition of the single stifle, blindly performed radiographic measurements.

Once all radiographic measurements have been completed, the blind was opened and cut-off values were identified, obtaining boundary thresholds between positive and negative results.

### Outcome parameters of diagnostic accuracy and statistical analysis

To identify the Δ_N_ cut-off between PA and clinically healthy stifles, the sensitivity was privileged identifying all the pathologic stifles avoiding false negatives. The Δ_N_ cut-off between HE and CO groups was identified giving equal weights to sensitivity and specificity, obtaining the cut-off using receiver-operating characteristic (ROC) methodology.

To carry out a validation of the translator device, as unit of analysis the single stifle was considered. Data from the three groups were compared calculating sensitivity, specificity, positive and negative predictive values, accuracy and ROC analysis with measurement of area under the ROC curve (AUC).

In order to evaluate thresholds and predictivity of Δ_N_ on groups, generalized linear mixed models (GLMM), with binomial distribution family and logit link, were used. In the GLMMs the “group” was used as fixed factor in order to explicitly quantify its effect, and “ID of the dog” was used as random factor, so addressing variability of stifles in the same dog and taking into account dependence of data. Predictions of the GLMMs were used to build ROC curves.

Specificity and sensitivity was used to evaluate the ability of the procedure to correctly identify pathological and healthy joints respectively; positive and negative predictive values were used to assess the probability to have a correct evaluation for a pathological and a healthy joint; accuracy was used to assess the proportion of observations classified correctly by the diagnostic procedure; AUC was used to summarize the overall performance of the diagnostic procedure.

In order to quantify the differences of Δ_N_ on the three groups, a linear mixed model (LMM) was fitted considering “group” and “DJD” as fixed factors and the “ID of the dog” as random factor to correct the statistical model taking into account the dependence of data. Post-hoc multiple comparisons of means were performed with Tukey contrasts test.

Potential within-dog association between joint instability (Δ_N_ values) and time since CrCL rupture (chronicity) or the presence of DJD, were evaluated with a linear model with “time in weeks” and “DJD” as predictors, together with their interaction. Significance of variables was obtained with family-wise confidence interval estimation.

A difference with a *P*-value ≤ 0.05 was considered statistically significant. R project for statistical computing, version 3.6.2, was used.

## Results

**Fig 2:**
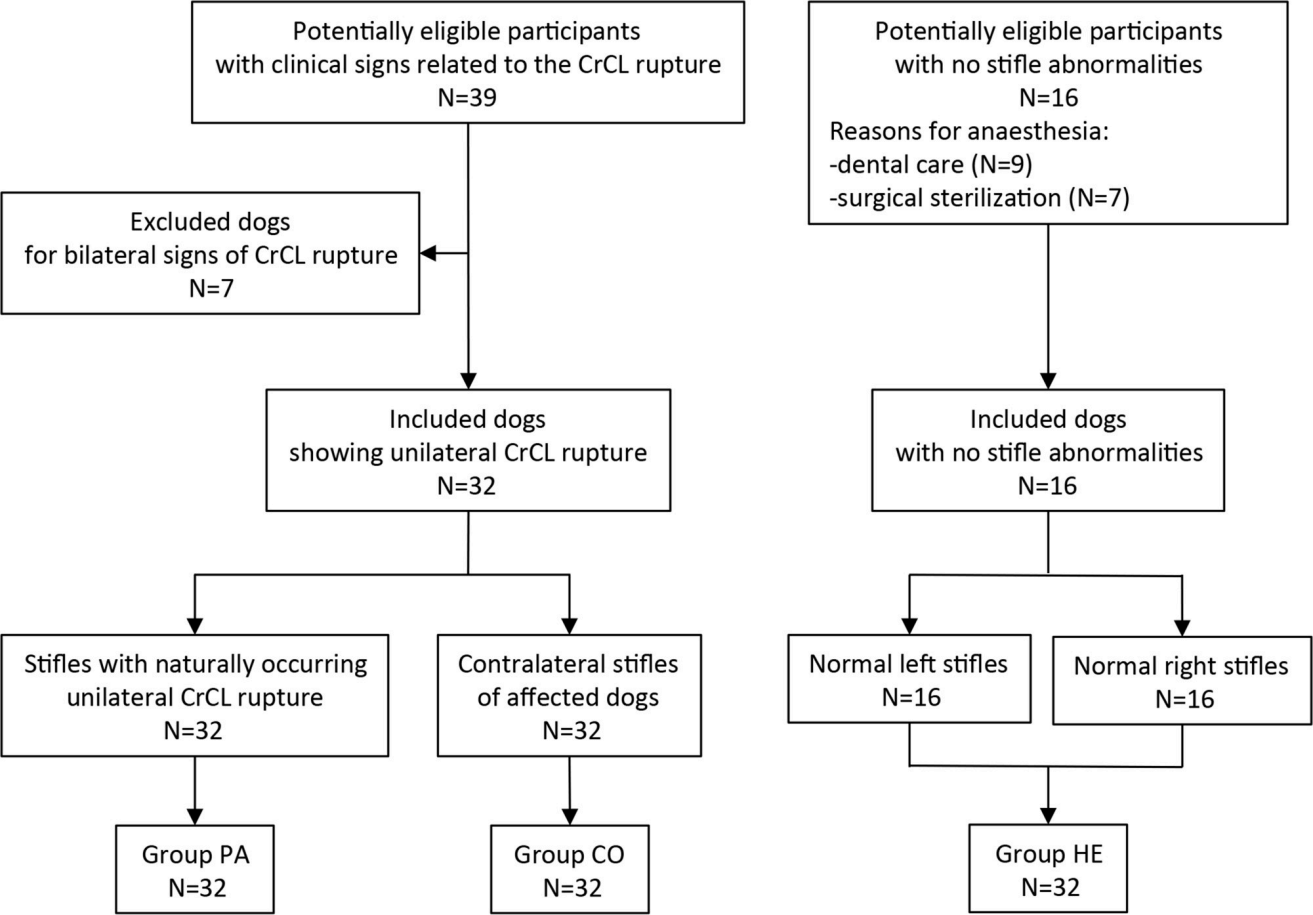
Flow of participants: STARD diagram.

A total of 96 stifles were included in this study (32 in Group PA, 32 in Group CO, 32 in Group HE) (Fig 2).

Population with unilateral CrCL rupture (Groups PA and CO) consisted of 17 males and 15 females; the mean age was 5.75 ± 2.02 years (range 2–11, median 5.5); the mean weight was 35.69 ± 11.59 Kg (range 15–57, median 36.5); the frequency of canine breeds represented in Groups PA and CO was: German shepherd and Rottweiler, 18.7% for each breed; Mix-breed, 15.6%; English bulldog, Épagneul breton, Labrador retriever and Pitbull, 6.2% for each breed; Alaskan malamute, Bullmastiff, Dogo canario, Golden retriever, Italian corso, Maremma shepherd and Leonberger, 3.1% for each breed.

Population of Group HE consisted of 8 males and 8 females; the mean age was 5.31 ± 2.27 years (range 2–10, median 4.5); the mean weight was 31.44 ± 10.56 Kg (range 16–51, median 29.0); the frequency of canine breeds represented in Group HE was: Mix-breed, 18.7%; Labrador retriever, Maremma shepherd and German shepherd, 12.5% for each breed; Boxer, Dobermann, English bulldog, Épagneul breton, Italian hound, Pitbull and Rottweiler, 6.2% for each breed.

The predictivity of Δ_N_ values resulted: highly significant on Group PA *versus* Group HE; statistically significant on Group PA *versus* Group CO; at the limit of statistical significance on Group HE *versus* Group CO (Table 1).

**Table 1:** Summary of the generalized linear mixed model (GLMM) assessing the predictivity of Δ_N_ values considering the “group” as fixed factor and the “ID of the dog” as random factor.

Groups	Variable	Estimate	Standard Error	*P*-value
**PA *vs* HE**	**Intercept**	-47.63	0.005319	<2e-16
	**Δ**_**N**_	1.873563	0.005313	<2e-16
**PA *vs* CO**	**Intercept**	-15.6694	4.8733	0.00130
	**Δ**_**N**_	0.5249	0.1602	0.00105
**CO *vs* HE**	**Intercept**	-29.006	14.685	0.0482
	**Δ**_**N**_	2.103	1.081	0.0516

Δ_N_: normalized tibial translation; PA: pathological stifles; CO: contralateral stifles; HE: healthy stifles.

The general results of the linear mixed model fitted to quantify differences of Δ_N_ on the three groups are summarized in the Table 2.

**Table 2:** General summary of the linear mixed model (LMM) considering the “group” and the “presence of DJD” as fixed factor and the “ID of the dog” as random factor.

Variable	Estimate	Standard Error	*P*-value
**Intercept**	12.220	1.466	9.95e-11
**Group CO**	7.951	1.960	0.000143
**Group PA**	40.824	2.523	2.e-16
**DJD**	-15.355	2.537	3.90e-08

PA: pathological stifles; CO: contralateral stifles; HE: healthy stifles; DJD: degenerative joint disease.

Significant differences between groups were observed in the LMM comparing Δ_N_ values (R^2^ = 84.67%, *P*<0.000). A large discrepancy in Δ_N_ values between HE (12.22±3.77%, range 5.90–19.00, median 11.12) and PA (43.59±12.97%, range 29.73–66.67, median 38.16) was observed (*P*<0.000). The Δ_N_ values of CO (20.32±6.69%, range 8.57–35.00, median 21.52) showed middle level between HE and PA stifles, while presenting statistical difference with both groups (*P* = 0.000125 and *P*<0.000 with HE and PA respectively).

In Group PA, 12 stifles did not show radiographic signs of DJD and 20 stifles showed mild degree of DJD; the average time since CrCL rupture (chronicity) was 6.31±3.58 weeks (range 2–16, median 6). The within-dog analysis of the association between joint instability (Δ_N_ values) and chronicity or presence of DJD shows that chronicity has relatively low significance in joint instability (*P* = 0.0691), and the biggest impact is from DJD (*P*<0.001). However, since the interaction is significant, chronicity effect is also relevant due to its combined effect with DJD (*P* = 0.0372) (Table 3).

**Table 3:** General summary of the linear model to assess potential within-dog association between joint instability and time since CrCL rupture (chronicity) or the presence of DJD.

Variable	Estimate	Standard Error	*P*-value
**Time**	-5.113	2.369	0.0691
**DJD**	-40.165	9.940	<0.001
**Time-DJD**	6.078	2.478	0.0372

Time: time in weeks since CrCL rupture; DJD: degenerative joint disease; Time-DJD: interaction Time and DJD.

**Fig 3:**
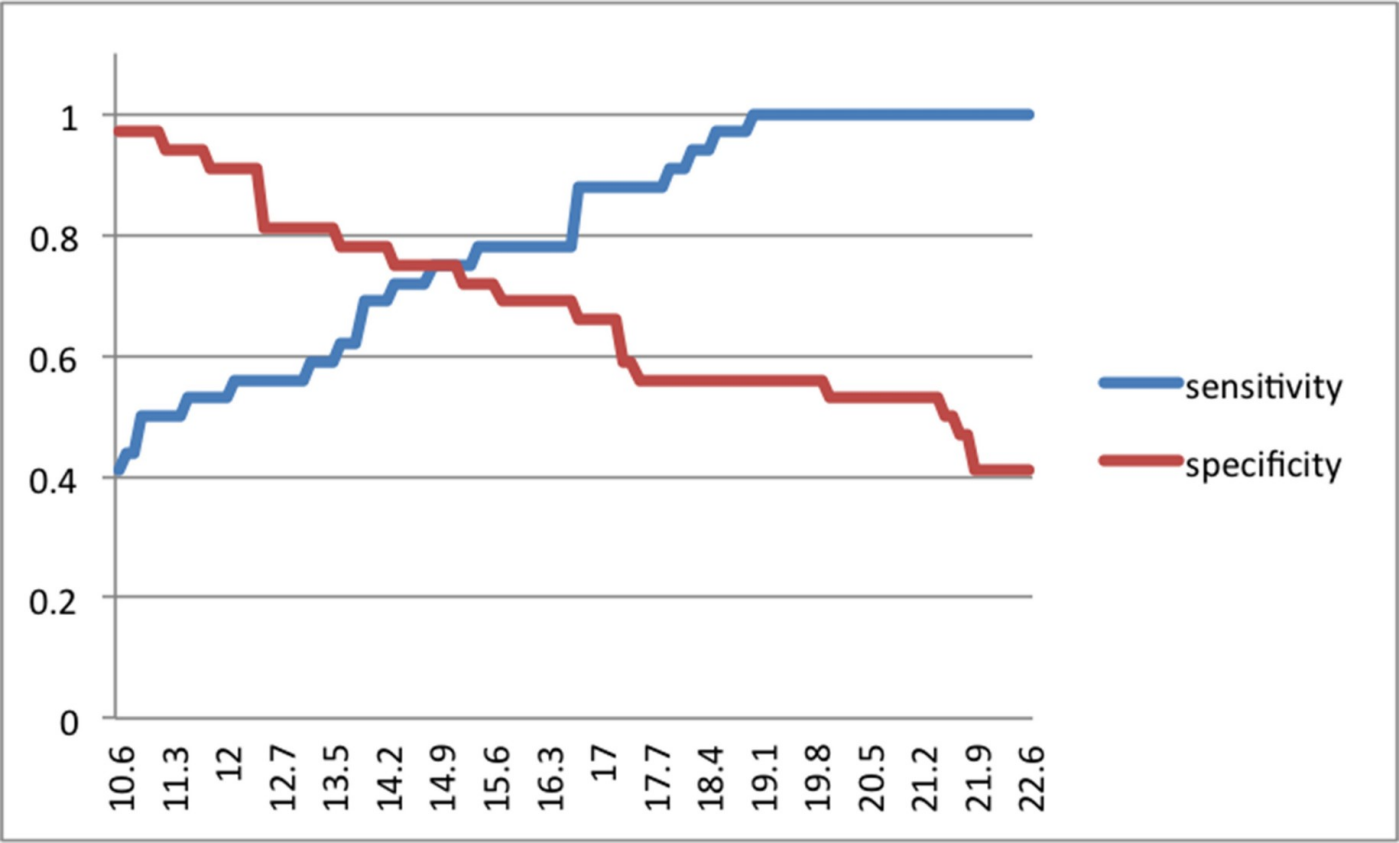
Two-graph ROC curve of the diagnostic sensitivity and specificity as a function of the test result (normalized tibial translation, Δ_N_, %) showing the Δ_N_ optimal cut-off between HE and CO groups.

The Δ_N_ cut-off value between PA and clinically healthy stifles resulted to be 29.73%. The Δ_N_ cut-off value between HE and CO stifles resulted to be 14.80% (Fig 3).

**Fig 4:**
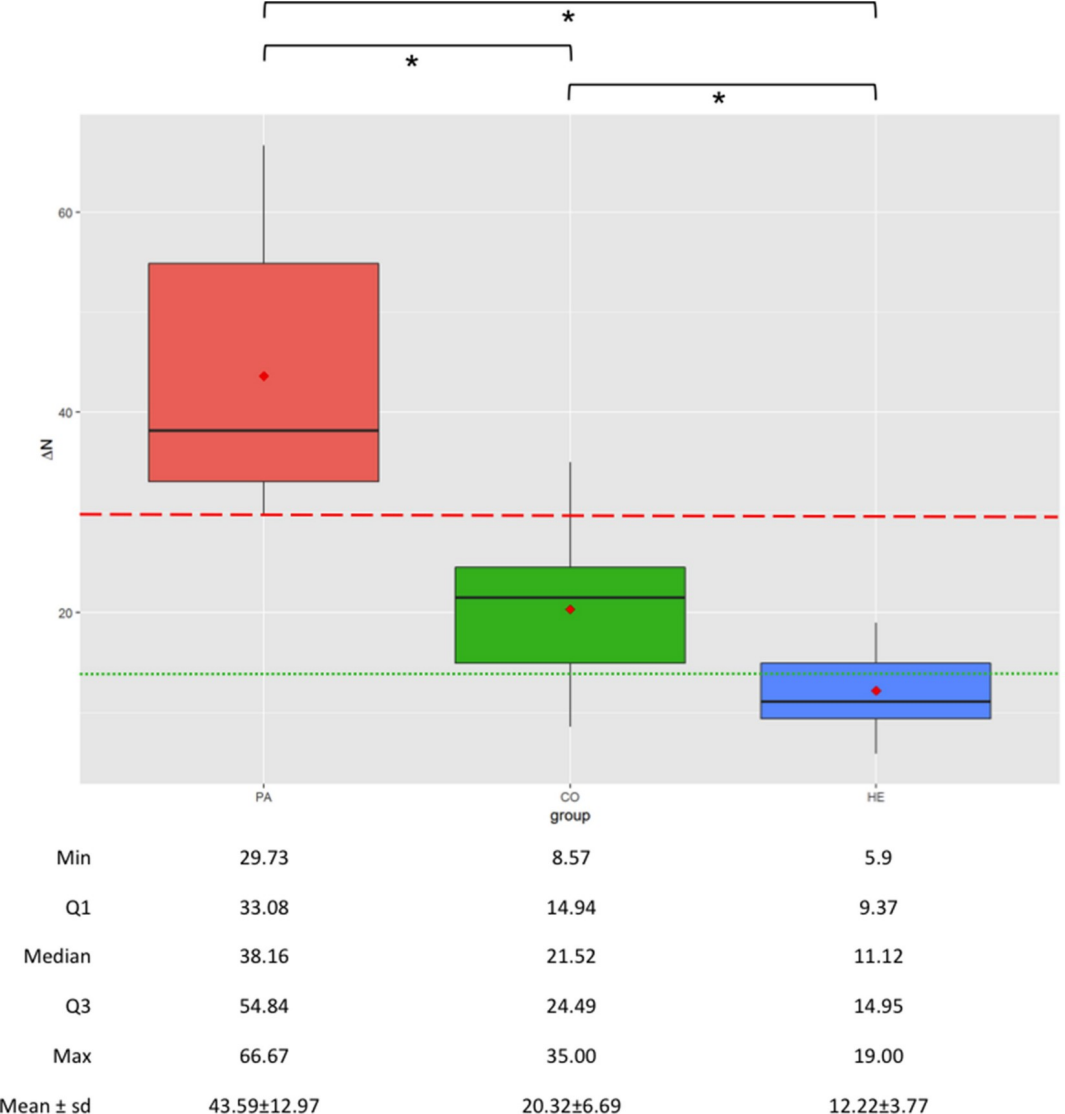
Boxplots showing the normalized tibial translation (Δ_N_, %) in groups PA (pathological, canine stifles with naturally occurring uni-lateral CrCL rupture), CO (contralateral stifles of affected dogs) and HE (healthy, canine stifles with intact CrCL); the ends of the whiskers show minimum (Min) and maximum (Max) values of Δ_N_; boxes show the median, the first and the third quartile (Q1 and Q3 respectively); red dots show the mean; dashed red line represents Δ_N_ cut-off between PA and clinically healthy stifles; dotted green line represents Δ_N_ cut-off between HE and CO groups; * indicates statistically significant differences.

The results of the Δ_N_ measured in Groups PA, CO and HE are summarized in the box-plot graph (Fig 4).

The results of the diagnostic validation of the translator device are summarized in Table 4.

**Table 4:** Validity assessment of the translator device.

Groups under comparison	PA *vs* HE	PA *vs* CO	CO *vs* HE
**Prob. thresholds**	0.499876	0.411041	0.4984901
**SE**	1.0 (0.999, 1)	0.9375 (0.816, 0.992)	1.0 (0.905, 1)
**SP**	1.0 (0.999, 1)	1.0 (0.991, 1)	1.0 (0.910, 1)
**PPV**	1.0 (0.999, 1)	1.0 (0.986, 1)	1.0 (0.985, 1)
**NPV**	1.0 (0.999, 1)	0.9412 (0.809, 0.957)	1.0 (0.985, 1)
**ACC**	1.0 (0.999, 1.0)	0.9688 (0.892, 0.996)	1.0 (0.944, 1.0)
**AUC**	100 (100, 100)	94.77 (86.41, 96.88)	100 (100, 100)
**ROC curves**	**S1 Fig**	**S2 Fig**	**S3 Fig**

Δ_N_: normalized tibial translation; Prob.: probability; PA: pathological stifles; CO: contralateral stifles; HE: healthy stifles; *vs*: *versus*; SE: sensitivity; SP: specificity; PPV: positive predictive value; NPV: negative predictive value; ACC: accuracy; AUC: area under the ROC (receiver-operating characteristic) curve; in brackets: 95% confidence interval.

No adverse events were recorded during or after the use of the translator device in radiographic evaluations. The entire radiographic procedure, including the premedication and preparation of dogs and the acquisition of radiographs in both stifles, was always completed within 30 minutes.

## Discussion

A validation assessment of the diagnostic method was performed to assess potential use in clinical trials.

With the systematic application of this procedure, using the achieved cut-off, a diagnosis of CrCL rupture could be issued with very high probability being certainly possible to discriminate (Accuracy and AUC) a healthy joint (Specificity) and an affected joint (Sensitivity) being all parameters at maximum levels.

Although both groups of stifles were considered not pathological, comparing HE with CO it was possible to obtain significant levels of sensitivity, specificity, positive and negative predictive values and accuracy, as also shown the high level of accuracy evidenced by the AUC method.

Measurements of Δ_N_ allowed to differentiate HE from PA and to accept the hypothesis that changes in CrCL integrity resulted in detectable changes on tibial translation.

In addition, a significant difference of Δ_N_ between CO and HE stifles was showed. The laxity of the CO stifles could be related to the high frequency of CrCL rupture in the contralateral stifles of unilaterally affected dogs [15–17,26,27].

The first cut-off value (29.73%) allowed identifying all stifles affected by CrCL rupture, maximizing the sensitivity at a slight expense of specificity. Few contralateral stifles exceeded this cut-off value; they did not show clinical cranial translation but showed a high Δ_N_ (false positives). In Group CO stifles, although showing higher radiographic translation than Group HE stifles, clinicians had the inability to palpate a cranial drawer sign. One potential reason for this finding is that the force applied manually by a clinician may not suffice to generate a cranial tibial displacement sufficient for clinical detection, as supposed by other author [28]. It therefore appears conceivable that, in our study, the absence of cranial drawer sign in Group CO reflected insufficient loading and lack of sensitivity of the cranial drawer test compared to the objective data generated during radiographic method testing. It could be hypothesized that the measured radiographic instability may be considered a potential high risk factor for CrCL rupture.

The second cut-off (14.80%), adopted getting a compromise between sensitivity and specificity, allowed the identification of stifles with low Δ_N_ and with low potential predisposition to CrCL rupture.

A potential limitation of the study is that affected stifles (Group PA) were confirmed by direct inspection of the damaged CrCL at arthrotomy during therapeutic surgery. Contralateral and healthy stifles (Groups CO and HE) were considered normal on the basis of clinical and radiographic examinations. However, because Groups CO and HE did not undergo CrCL inspection for ethical reasons, there is a possibility of false negatives or partial CrCL tears in these groups, and this could be considered a potential source of bias, which could potentially influence the analysis of diagnostic accuracy of this radiographic method.

A number of *ex vivo* biomechanical studies assessed the ability of several surgical techniques to control joint stability after cruciate ligament rupture [25,28,29]. This suggests that the evaluation of the degree of joint stability could be considered of crucial importance in clinical cases.

The translator device could be considered a simple, easy-to-use, inexpensive and useful tool to objectively quantify the *in vivo* cranial tibial translation in dogs with CrCL rupture. The procedure could be used in veterinary clinical practice to evaluate canine stifle stability; nevertheless, it could be particularly useful in clinical trials by including it among objective evaluation protocols to be used before/after surgery and during post-operatory follow-up. It could be used in clinical trials evaluating intra-articular or extra-articular stabilization techniques. Although it has not been investigated in this study, the procedure may be apparently less indicated for tibial osteotomy techniques (e.g. TPLO, TTA), as they purportedly tend to restore dynamically the biomechanics of the joint [12,30,31]. However, the still not clear scientific knowledge and the growing development of research in this field [12,29–33] could warrant further studies using the device even before/after osteotomy techniques.

Further long-term follow-up retrospective study will be necessary to confirm the hypothesized predictive value for CrCL rupture in dogs.

## Supporting information

S1 Table STARD checklist. (PDF)

S1 Fig ROC curve of normalized tibial translation (Δ_N_) considering Group PA (pathological stifles) and Group HE (healthy stifles). (PDF)

S2 Fig ROC curve of normalized tibial translation (Δ_N_) considering Group PA (pathological stifles) and Group CO (contralateral stifles). (PDF)

S3 Fig ROC curve of normalized tibial translation (Δ_N_) considering Group CO (contralateral stifles) and Group HE (healthy stifles). (PDF)
