# Supplementary material for: Evaluation of cranial tibial translation in dogs: Diagnostic accuracy of radiographic method using a simple device
Source: PLoS One. 2020 Feb 11;15(2):e0228621. doi: 10.1371/journal.pone.0228621 (PMC7012417; doi:10.1371/journal.pone.0228621)
Supplement: S2 Fig — (PDF) [file pone.0228621.s003.pdf]

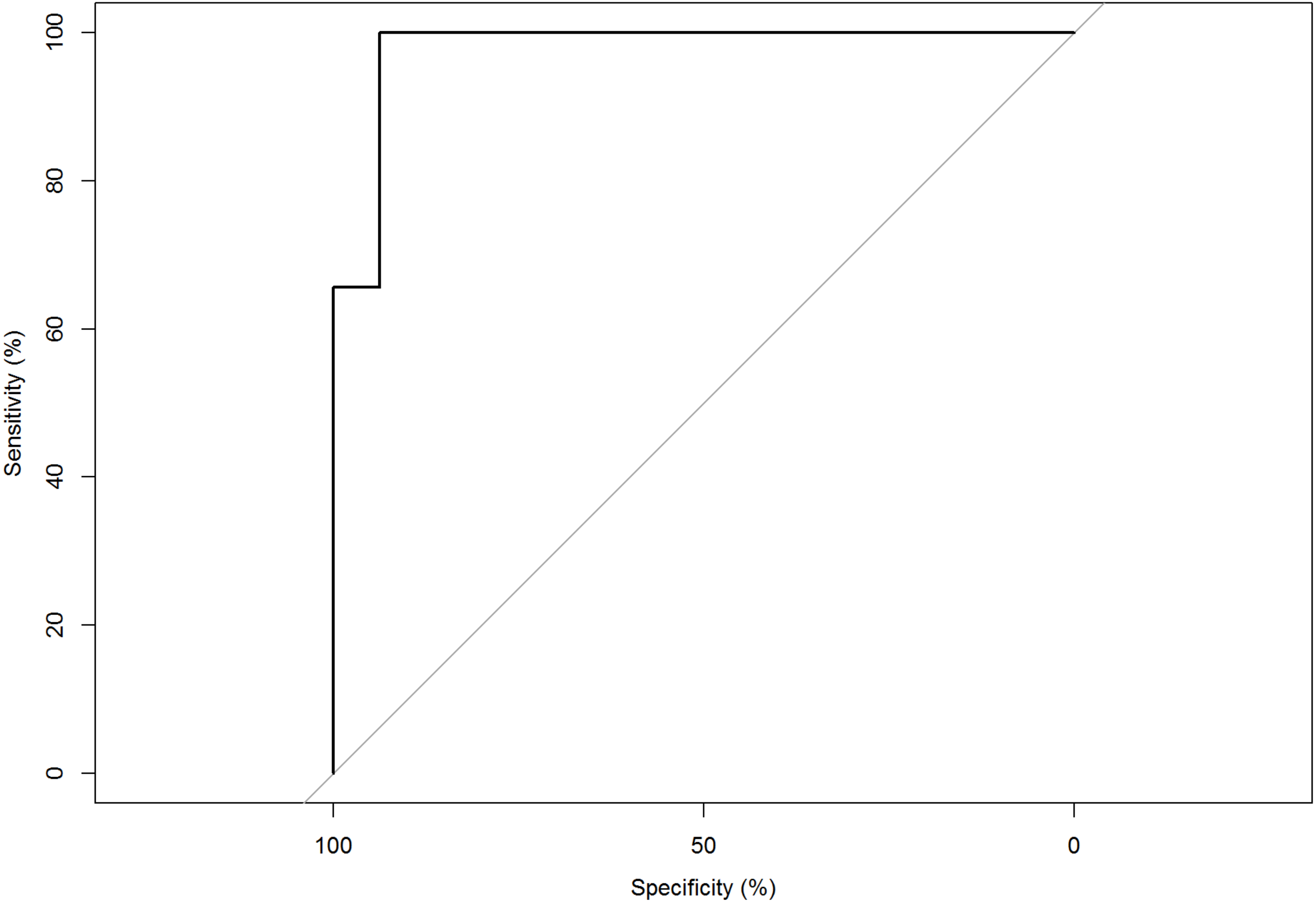

S2 Figure. ROC curve of normalized tibial translation (Δ_N) considering Group PA (pathological stifles) and Group CO (contralateral stifles).
