# Supplementary material for: Evaluation of cranial tibial translation in dogs: Diagnostic accuracy of radiographic method using a simple device
Source: PLoS One. 2020 Feb 11;15(2):e0228621. doi: 10.1371/journal.pone.0228621 (PMC7012417; doi:10.1371/journal.pone.0228621)
Supplement: S3 Fig — (PDF) [file pone.0228621.s004.pdf]

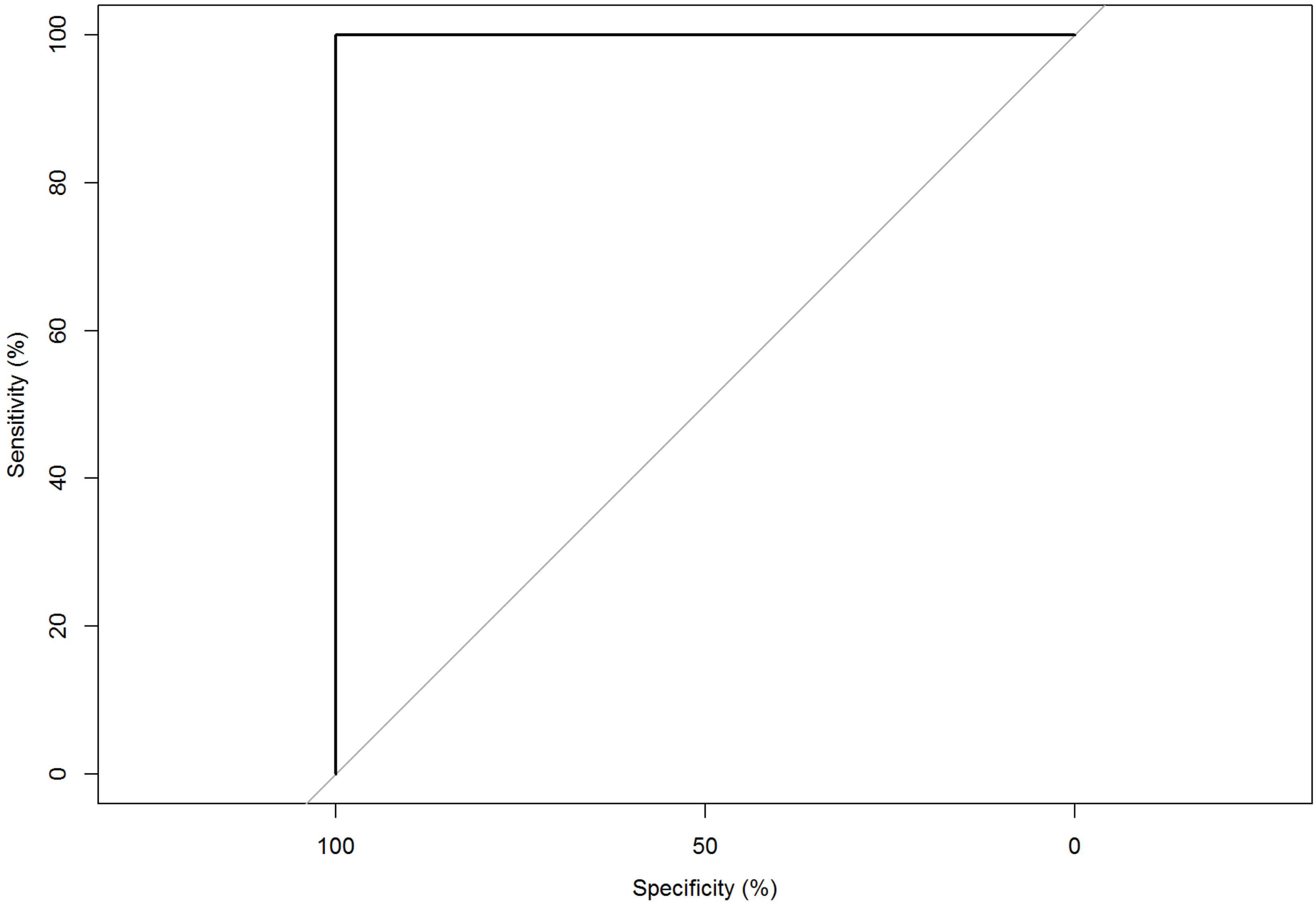

S3 Figure. ROC curve of normalized tibial translation (Δ_N) considering Group CO (contralateral stifles) and Group HE (healthy stifles).
